# Designing Antennas for RFID Sensors in Monitoring Parameters of Photovoltaic Panels

**DOI:** 10.3390/mi11040420

**Published:** 2020-04-17

**Authors:** Mariusz Węglarski, Piotr Jankowski-Mihułowicz, Mateusz Chamera, Justyna Dziedzic, Paweł Kwaśnicki

**Affiliations:** 1 Department of Electronic and Telecommunications Systems, Rzeszów University of Technology, Wincentego Pola 2, 35-959 Rzeszów, Poland; mateusz.chamera@talkinthings.com; 2 Talkin’Things, Al. Wilanowska 317, 02-665 Warsaw, Poland; 3 ML SYSTEM SA, Research & Development Centre for Photovoltaics, Zaczernie 190 G, 36-062 Zaczernie, Poland; justyna.dziedzic@mlsystem.pl (J.D.); kwasnickipawel@gmail.com (P.K.)

**Keywords:** RFID (radio-frequency identification), passive transponder, semipassive tag, energy harvesting, RFID sensor, Internet of Things (IoT)

## Abstract

The importance of the radio-frequency identification (RFID) technology and photovoltaic (PV) systems has been growing systematically in the modern world full of intelligent products connected to the Internet. Monitoring parameters of green energy plants is a crucial issue for efficient conversion of solar radiation, and cheap RFID transponders/sensors can be involved in this process to provide better performance of module supervision in scattered installations. Since many components of PV panels disturb the radio-wave propagation, research in the antenna scope has to be carried out to reach the proposed fusion. The problem with RFID transponders being detuned in close proximity to glass or metal surfaces can be solved on the basis of solutions known from the scientific literature. The authors went further, revealing a new antenna construction that can be fabricated straight on a cover glass of the PV panels. To achieve the established task, they incorporated advantages from the latest advancements in materials technology and low-power electronics and from the progress in understanding radio-wave propagation phenomena. The numerical model of the antenna was elaborated in the Hyper Lynx 3D EM software environment, and test samples were fabricated on the technology line of ML System Company. The convergence of calculated and measured antenna parameters confirms the design correctness. Thus, the studied antenna can be used to elaborate the cheap semipassive RFID transponders/sensors in the PV panel production lines.

## 1. Introduction

### 1.1. RFID Technology

The radio frequency identification (RFID) technology is often used in automated processes in various areas of human socioeconomic activity [1]. Its usefulness is confirmed by the growing number of new applications [2,3,4]. RFID devices are increasingly used in sectors such as security and access control systems, logistics, trade in fast-moving consumer goods (FMCG), research experimental samples and valuable materials in the world supply chains [5]. Important applications can be also found in the automatic vehicle identification (AVI) area, including reliable and safe object identification in rail or road transport along with the tracking and navigation of moving vehicles [6]. It should also be noted that the RFID technology is becoming more and more common in distributed sensor networks, in which it is used to monitor operation parameters of various types of installations. These implementations have important meaning for creating the Internet of Things (IoT) [7]. In economic terms, this is due to the increasing availability of RFID equipment on the market, especially new constructions of transponders based on intelligent semipassive chips with additional functionalities (such as power harvesting or physical quantity measuring capabilities) [8]. In technical terms, the good forecast in this area in the coming years is due to progress in recognition of the operational principles of RFID devices and the possibilities of determining their parameters [9,10]. 

**Figure 1:**
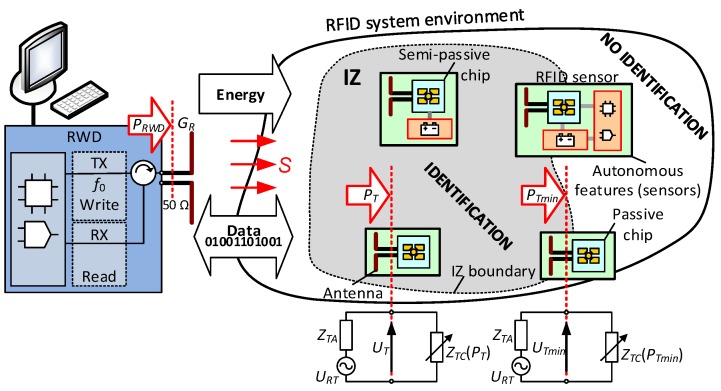
General diagram of an ultrahigh-frequency (UHF) radio-frequency identification (RFID) system. The interrogation zone (IZ) is space in which communication process can be carried out and thus where the unique identification number (UID), user data and measured parameters can be read (or written with user data).

Regardless of frequency parameters (operating frequency *f*_0_ and frequency band: low (LF), high (HF) or ultrahigh (UHF)), world regulation of the radio-transmissions (e.g., ISO/IEC 15693, 18000-63 [1,11]) and standardized system implementations (e.g., HITAG, MIFARE and ICODE; NXP Semiconductors, Eindhoven, The Netherlands), the read/write device (RWD) and one or several transponders with a unique identification number (UID) attached to identified objects can be always found in every RFID system (Figure 1). Application efficiency in the automatic identification field is mainly described by the three-dimensional interrogation zone (IZ) [12]. The IZ is the main parameter that allows to predict the usefulness of the RFID system in real conditions. Nevertheless, it is very hard to estimate this parameter outside the laboratory site; therefore, the read range (read/write range) is mostly determined [13,14] in practice, generally by the “trial and error” method that is the most commonly used in the industry [15,16]. In the presented research, indeed, particular attention is paid to the construction of an electronic transponder, but nevertheless the origin of the undertaken efforts lay in the desire to improve the process of predicting the real IZ on the basis of determined parameters at simulation and design stages. 

The most popular type of RFID passive transponder consists of two main elements: an antenna and a chip (IC—integrated circuit) [1]. If there is an additional supply source (typically a disposable lithium battery but also a replaceable accumulator) in its construction, then such a device is called a semipassive transponder. The main purpose of providing the extra energy source is the need to enlarge the IZ or to execute additional functions (such as data acquisitions, supervising environment, controlling actuators) without an active RWD [17,18]. Such implementations are possible since the RFID chips are provided with an extended memory for measured samples and external wired interfaces for data exchange with other microcontroller systems. It should be noted that, for conducting radio communication in RFID systems, the RWD has to actively send queries, and then RF frontends of transponders are powered by energy passed through the electromagnetic field. In this way, the additional source can only help to improve parameters achieved in the established link. The antennas of both passive and semipassive tags do not emit an electromagnetic field at all. They only influence the field when the input impedance of the chip is keying. This method of passing signal back to the interrogator is called the backscattering. This property allows to distinguish the RFID transponders from well-known short-range devices (SRDs) [19].

### 1.2. Princeples of Photovoltaic Panels

A typical photovoltaic panel (module) is the basic element of a photovoltaic power plant, and it contains a few (or dozens of) cells, depending on generation, design, maximum power, nominal voltage, etc. [20]. The cells and then modules are usually connected in series to reach the nominal voltage. Then, the chains are joined in parallel to achieve the expected power for supplying, e.g., a building, or for passing it to the electrical distribution grid. 

**Figure 2:**
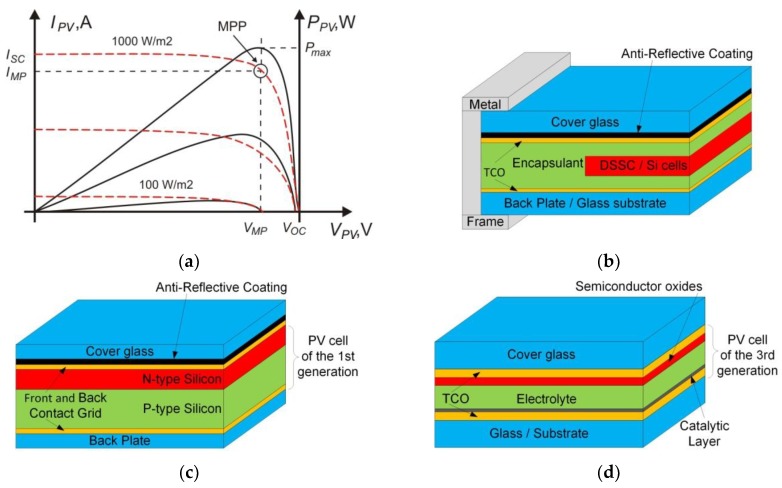
Photovoltaic (PV) panel. (**a**) I-V and P-V characteristics of a photovoltaic panel at solar irradiation from 100 to 1000 W/m^2^; *I_SC_*—short-circuit current (at *V_PV_* = 0); *V_OC_*—open circuit voltage (at *I_PV_* = 0); *V_MP_* and *I_MP_*— voltage and current, respectively, at maximum power point (MPP). (**b**) Common structure of exemplary PV panel. (**c**) Common structure of PV cell of the first generation (Si—silicon). (**d**) Common structure of PV cell of the third generation (e.g., DSSC—dye-sensitized solar cell).

The most important parameters of the PV panels (or cells) are the current–voltage and power–voltage nonlinear characteristics (Figure 2a) [21,22]. On the basis of the curves, further parameters can be determined, such as the short-circuit current (*I_SC_*) that occurs when the voltage across the solar cell is zero (*V_PV_* = 0) as well as the open-circuit voltage (*V_OC_*) measured at open terminals of the cell (*I_PV_* = 0). When the module is operating under load conditions, it should work at the maximum power point (MPP), which is available at the bend of the characteristics (*V_MP_* and *I_MP_*, which are the voltage and current, respectively, at MPP)—it is the transition from the state with the constant voltage to the steady current. All the parameters allow to select the optimal operating state for power installation in different conditions of current load, solar irradiation and temperature. It should be noted that they are not easily determinable, especially with regard to the module that is installed in the target system. The crucial issue in the PV plant with a multitude of modules connected in series and rows is to find the best maximum power point. When every panel works with the same I-V characteristic and under the same irradiation and temperature, the problem of tracking MPP can be easily solved, and standard PV converters are equipped with such a function [23,24]. However, some modules of the power matrix may disturb this process of power point finding because of local shading occurrence, nonuniform temperature distribution, contamination of active surface, uneven degrees of element aging, etc. Generally, such problems influence the current carrying capacity, but when *I_PV_* is lower than *I_MP_* the problems may not be noticed. On the other hand, panel overloading reduces the cell performance and leads to a significant voltage drop at the panel terminals; in some cases, the voltage can even change the polarity (which is prevented by protective diodes). In a large matrix, several local extremes can also appear in the P-V curves. These all may significantly disrupt and, in consequence, reduce the volume of energy production. Therefore, the best solution, but a very expensive one, would be to equip all individual panels with dedicated DC–DC converters with the function of maximum power point tracking. Since such a construction is not used (because of costs), it is necessary to monitor parameters of all modules, otherwise one weak cell can destroy the efficiency of the entire installation. 

In order to know the exact condition of a particular panel, at least the current, voltage, solar irradiation and temperature should be measured or estimated in any other way. If a comparative method was used, the measurement task could be simplified. Assuming that all panels work in the same conditions—meaning that they are loaded with the same normal current for series connection, that they are uniformly irradiated and that the temperature is similar on all devices—they should generate the same voltage at the terminals. Thus, in order to indicate a degraded, damaged or odd panel, it is sufficient to measure the voltage at a given current in the chain and to relate the results to the I-V characteristic at a given temperature and irradiation. Obviously, the acquired data have to be synchronized in the time domain. It is important that the odd panel can be distinguished without having to invade the structure of connection in the PV system, as this is very troublesome and has to be performed by qualified personnel. 

Various acquisition systems for monitoring the parameters of PV panels have been designed and described in the related literature [25,26]. All of them work being supplied by photovoltaic energy. The authors propose in the paper to use the ideas derived from the RFID technology, particularly from constructions dedicated to being implemented in the RFID sensors. The main advantage of this solution is that the acquired data are always accessible, even if the energy system collapses. Furthermore, the electronic transponders can be used at every life stage of the PV devices. 

A typical PV panel consists of several layers (Figure 2b). In general, the glass covers the sunny side, providing protection for the internal structure against external interferences and degradation under the influence of the environment, while allowing maximum transmission of the solar spectrum. Such a construction is characteristic for every generation of PV panels: the monocrystalline and polycrystalline silicon are used in the first generation (Figure 2c); the thin-film technology is used in the second generation; the semiconductor junctions are replaced with active elements based on other physicochemical phenomena that allow to convert the solar energy into electricity in the third generation (Figure 2d). The glass cover is often modified with a conductive metal layer such as a transparent conductive oxide (TCO) glass, and the net of connections between cell electrodes is made with the screen-printing technology on its surface [20]. 

### 1.3. Issue of PV Panel Monitoring by RFID

The applicability of the RFID technology in the photovoltaic installations can be considered in two aspects. Primarily, the RFID transponder can serve as an electronic tag that provides detailed information about an object (this is the panel) at all stages of its life: production, distribution (in the supply chain), installation (in the target application), operation, service/maintenance and utilization. Introducing such a service could significantly improve logistics and maintenance of products, as it would not be necessary to use other identification systems. Nowadays, RFID systems are usually rarely used on production lines, whereas they often appear at the stages of distribution and trade. This means that the transponders have to be attached to the product, intentionally, for only these two intervals of their life. Moreover consumers cannot use this facilitation in their household. On the other hand, the modern RFID technology provides the ability to modify data in the transponders’ internal memory in accordance with consumer requirements. Intelligent implementations can even learn about the environment by gathering data with a variety of sensors embedded in the structure of the sempassive RFID transponders [27,28]. This feature, which is still not used in automated identification systems, has been implemented in the presented application of PV panel supervising. 

The parameter monitoring of individual panels is the key enhancement that can improve efficiency of the photovoltaic plant. Since the monitoring devices are designed as low-power technology, they can be supplied by electricity produced in the PV cells; in the electric plant, this minute load is imperceptible. Nevertheless, the voltage at the panel terminals is not available at night, which is a significant problem when the SRDs need to be used. The problem can be solved by equipping the parameter monitors with energy accumulators (e.g., supercapacitor in the simplest version or a chemical cell when maintenance is anticipated). The capacious storage element is expensive and bulky, while the little one is not able to supply circuits that emit electromagnetic signals. Thus, transmitters in SRDs rather do not respond when energy is restricted and especially when the panel is damaged. Furthermore, when the economic requirements (such as being low-cost, maintenance-free and easily integrable) were considered, the use of the RFID sensor was determined as the best choice to solve all the above-mentioned problems. Full integration of passive and semipassive transponders with marked objects is a desirable feature sought nowadays in implementations of the RFID technology. The auxiliary power source is not necessary to read or write the tags’ memory because of the passive nature of the RF transmitter/receiver. Since the transponders are supplied by the electromagnetic field generated by the RWD antenna, measurement tasks can also be achieved when the PV panels are not active. 

In consideration of the listed benefits of RFID and PV technological fusion, as well as the need to obtain the largest interrogation zones, the assumption of applying RFID systems operating in the UHF band has been established. The frequency range of 860–960 MHz has been assumed in order to cover all standards regardless of the type of equipment used, whose operating parameters may differ depending on the standards existing in various places around the world. The long-range RFID systems operate in accordance with the requirements of the electronic product code EPC global gen. 2 [29] normalized by ISO/IEC 18000-63 [11]. The European version of these systems assumes compliance of read/write devices and their antennas with ETSI EN 302 208 [30], where power is limited to 2 W ERP (effective radiated power) in the frequency band of 865.6–867.6 MHz. In the FCC Part 15.247 American standard, the limit is 4 W EIRP (effective isotropic radiated power) at 1 W output power of the transmitter and an antenna with a maximum gain of 6 dBi, in the frequency band of 902–928 MHz.

The design solutions of the measuring acquisition system are dependent on the accessibility of appropriate electronic components, advancement of monitoring model, quantity of gathered data and assumed scenarios for supervising the PV panels. In the simplest case, it is possible to use only additional blocks that are included in commercially available RFID chips. Further simplification could be achieved if the concept of measuring only voltage at the panel’s terminals was considered. Such an RFID sensor could be activated just by a query from the RWD only during the service routine (both related to periodic inspections or fault detections), and the measured values received from the transponder might be processed in a management center of the RFID system. The comparison of the voltage on adjacent panels in the power chain (at known temperature, solar irradiation and I-V curve) gives enough information to carry out the effective diagnosis. This embodiment is the cheapest and completely corresponds to the most important goal, which is to quickly and clearly identify the damaged panel. On the other hand, the most complex solutions have to be designed on the basis of microcontrollers for conducting measurement tasks, data processing and transmitting data via wire or straight through radio interfaces as well as for the advanced energy managing. Of course, such an entire circuit can be integrated into a one semiconductor chip with the option of connecting external transducers of physical quantities, but this requires significant financial resources. All the above-mentioned aspects of designing RFID sensors that are possible to be adopted in the monitoring of PV panels are discussed in detail in [27]. 

### 1.4. Integration of Antennas and PV Panels 

Apart from the issue of developing electronic circuits of RFID sensors, which is revealed in [27], the most difficult problem consists in designing an antenna that can cooperate with the semipassive chip with the extended internal structure of additional functionalities and could be attached to the PV panel without losing its effectiveness. The enlargement of the interrogation zone is limited mainly by the energy conditions that have to be satisfied in the UHF RFID system [1] and particularly by the possibility of passing energy to the transponder in the presence of photovoltaic cell components that disturb the electromagnetic field. Therefore, it is very important to carry out the full process of antenna synthesis, in which the main focus is paid to maximizing the energy transfer to the RFID chip. It should be emphasized that placing the antenna on the surface or in the close vicinity of the photovoltaic cell requires the use of special constructions of inductive circuits (including additional reflectors under the radiators). Generally, the concepts of designs dedicated to operate in the harsh environment or to be attached to metal surfaces can be utilized to create new implementations in this scope. The reason for this is the presence of metal-oxide layers and fluids (electrolyte in third-generation cells), as well as metal electrodes in the PV panels, that strongly affect the impedance parameters of inductive and capacitive circuits and thus the efficiency of energy transmission in the RFID systems. Even when the transponder is placed on or integrated with the cover glass, the material parameters of all components in the PV module have to be taken into account in the synthesis process [31]. 

Common transponders are developed to be located on surfaces or in structures that are mainly based on plastics or textiles. In addition, designers strive to reduce dimensions of both antennas (e.g., by replacing dipole antennas with meandering or additional arms as well as designing microstrip patch structures) and chips/ICs (e.g., reducing the channel length of transistors) which often decrements the read/write range. They develop antennas with an omnidirectional radiation pattern in order to diminish the sensitivity to transponder deviation against the RWD antenna [32]. If such a tag is placed on a conductive surface or glass with TCO, the antenna efficiency is significantly attenuated due to a change in its impedance and resonance circuit detuning. Therefore, the most important factor in proper antenna synthesis is achieving the best impedance-matching with the highly capacitive RF frontend of the chip. 

Of course, the issue of designing antennas for electronic transponders is discussed in many scientific publications, as this is a key element that determines the effectiveness of the entire RFID system. Nevertheless, new constructions dedicated to specific applications are still being developed taking into account the whole UHF band and sensitivity to manufacturing inaccuracies and various environmental conditions. 

## 2. Materials and Methods 

### 2.1. Impedance Matching

The authors consider the RFID systems of the UHF band in their work. Furthermore, devices operating in the far field are assumed to be the best choice for designing the desired application. This means that the electromagnetic wave generated in the antenna system is considered locally as a plane wave in which strength vectors of electric and magnetic fields are perpendicular to each other and also to the direction of the wave propagation. Energy is carried between the matched antennas of RWD and transponders by the radiated wave with a power density of *S* (Figure 1) and at an operating frequency of *f*_0_. However, it should be noted that the classically understood impedance matching (e.g., considered as resistance of 50 Ω) between a transmitter and receiver is only valid for the case of the RWD and its antenna. The input impedance of the chip varies with the power harvested in the transponder. 

The changes in the input impedance *Z_TC_* (Figure 1) of the RFID chip significantly complicate the design process of the transponders’ antennas. The impedance is expressed by a complex number and is related to the operation of the rectifier and stabilizer in the power harvester of the chip. Its value varies depending on the parameters of the electromagnetic field existing in the place of the tag location, on mutual arrangement of antenna system, i.e., the orientation of the tag according to the RWD antenna and its localization in relation to other objects that can disturb the radio-wave propagation. When the transponder is in the IZ, this means that the energy and communication conditions are met and that the minimum voltage *U_T_* that is induced at the antenna terminals activates the RF transmitter in the chip. However, the transmitter does not emit any energy—data from the tag to the RWD is sent by using the backscatter communication. In this process, the carrier wave is partially reflected (towards the RWD) using modulation of the chip input impedance. The communication rules are implemented in the protocol of electronic product code [29] that is standardized by ISO/IEC 18000-63 (formerly ISO/IEC 18000-6) [11]. 

Another key issue when designing the RFID transponders is to meet supply power requirements [33]. The voltage induced in the transponder antenna while it is located in the electromagnetic field generated by the RWD antenna is represented by the source *U_RT_* (Figure 1). The energy transmitted to the chip is represented by the voltage *U_T_* induced at the antenna terminals and by the power *P_T_*. The electronic circuit in the tag starts working at the minimal power *P_Tmin_*, known as the chip sensitivity. The value of the *P_Tmin_* is dependent on the type of chip. Primarily, this depends on whether the chip is passive or semipassive, but it also depends also on the used communication protocol, the manufacturer, the load generated by additional functionalities, energy support from additional sources, etc. In turn, the chip sensitivity influences further parameters such as the chip impedance at the IZ boundaries, the antenna construction and the shape and size of the interrogation zone in a given application. 

The most important determinant that indicates the correctness of the antenna design is to achieve the matching between the chip impedance *Z_TC_* and the antenna impedance *Z_TA_* [9], which is characterized by the power transfer coefficient *τ*:

(1) τ=4Re(ZTA)Re(ZTC)Re(ZTA+ZTC)2+Im(ZTA+ZTC)2

The antenna impedance has inductive nature, whereas chip impedance is capacitive; they are described by dependences as follows:

(2) $$Z_{TA}=R_{TA}+jX_{TA}=R_{TA}+j\omega L_{TA}$$

(3) $$Z_{TC}=R_{TC}+jX_{TC}=R_{TC}+\frac{1}{j\omega C_{TC}}$$

where *R_TA_* and *X_TA_* denotes resistance and reactance of the transponder antenna, respectively; *L_TA_* indicates its inductance; *R_TC_* and *X_TC_* indicate resistance and reactance of the active chip, respectively; *C_TC_* indicates its capacitance; and *ω* = 2π*f*_0_ describes pulsation.

It should be also taken into consideration that the impedance *Z_TA_* is constant at a given frequency, but the chip impedance *Z_TC_* varies while the transponder is working because of changes in power load generated by its electronic circuit. Both impedances are only coupled when the chip works at *P_Tmin_* and at the environmental conditions assumed in the designing stage. Otherwise, they are also strongly influenced by ambient conditions, such as the type of surface to which the tag is attached, temperature, humidity and vicinity of other objects. 

### 2.2. Selection of Antenna Construction for the Given Application

Many constructions of the transponders dedicated to the UHF band are designed as a modification of a dipole antenna with a linear polarization. Nevertheless, they are sensitive to environmental impacts, and thus any change in the dielectric permittivity of substrate influences the antenna properties [34]. Since maximization of the interrogation zone is the most important issue when considering the development of PV installation with a monitoring system based on the semipassive RFID transponders/sensors, it is necessary to consider other solutions. The antenna constructions with a circular or elliptical polarization and the omnidirectorial radiation pattern significantly enlarge the IZ. On the basis of the review of relevant literature, it should be stated that the fulfillment of these assumptions may be difficult, especially in relation to the given photovoltaic application. 

The issue of classical antenna (50 Ω) integration with the PV cells has been raised [35]. The developed constructions are dedicated for operating in the upper part of the UHF band (above 2 GHz). A microstrip patch antenna and a half-wave dipole antenna with a poly-Si solar cell as a reflector are considered by the authors. The antennas are dedicated for communication purposes, e.g., in future mini- and microsatellites, in sensor networks, wireless networks, mobile phones, and GSM or GPS systems. The authors want the solar cell to be an energy source as well as to play the role of radiating and receiving signal antenna. The conjunction of PV cells and microwave antennas significantly increases the range of advantages in terms of costs, occupied space, installation troubles, etc. The antenna manufacturing process involved the PCB technology. The authors also proposed a square microstrip patch antenna made on a Perspex substrate as an AgHT-4 film layer on a glass.

In some research papers, authors propose the RFID antennas with different shapes of radiation patterns that are dedicated to operate on various substrates (e.g., on glass [32], metal [36], plastic [37], paper [38]). However, due to the radiator shape, they cannot be implemented in the developed PV application, in which analog signals have to be connected to the extended circuit of the acquisition module. Designers planned a place inside the radiator only for a common RFID chip that just has pads for connecting the antenna. 

There is also research in which the RFID antenna constructions dedicated to work on glass surfaces are considered. For example, in [32] authors propose a circularly polarized antenna of a square-ring shape that can cooperate with the passive chip: Higgs-2 (Alien Technology, San Jose, NC, USA). Transponders of designs like that dedicated to operate in harsh environmental conditions such as close proximity of metal [36] are suggested to be installed on a car windshield by authors of [39,40]. A typical construction developed to operate on metal surfaces is presented in [36]. It is a rectangle antenna with a star-shaped slot and a terminal-grounded L-shaped feed-line that has circular polarization and is connected to the chip: Monza 4 (IMPINJ, Seattle, WA, USA). A special reflector is often used in order to reduce the environmental impact on proposed transponders. In another example, an F-shaped dipole antenna with an inset parasitic strip along a closed loop and matched to the passive chip, Higgs-2, is described in [40]. A more sophisticated solution is revealed in [39]: a dual-band antenna for the HF (13.56 MHz) and UHF (920–925 MHz) bands and HF SIC5600 (Silicon Craft Technology, Bangkok, Thailand) and UHF HiRead-T (NXP Semiconductors, Eindhoven, The Netherlands) passive chips. All the above-mentioned transponders are fabricated in the PCB technology on the FR4 glass-reinforced epoxy laminate. In [41], instead of the FR4, a thin PET substrate is used as a carrier for a copper antenna. The antenna is comprised of a trapezoidal matching loop and a dual-dipole radiator and is dedicated to the passive chip Monza 3 (IMPINJ, Seattle, WA, USA). A specific solution for fast-moving consumer goods (FMCG) is also available for glass products such as bottles with liquids [34]. The antenna is designed as a wire loop matched to the Monza 4. Although it has very good radio communication properties and is not very sensitive to liquid content, it is relatively large and is not fully integrated with the bottle. 

Detailed considerations regarding the energy budget in the UHF RFID system are presented in [42]. In this application, the passive transponder is mounted on a car windshield with a graphite polarized film. Such a substrate structure affects the antenna impedance and, in consequence, decreases the read/write range. On the basis of conducted experiments, the authors propose an antenna that can be matched to the Higgs-3 (Alien Technology, San Jose, NC, USA) passive chip when it is located on the windshield. 

Thus, it is possible to create RFID transponders that can operate on glass even though it significantly influences the antenna impedance value. In addition, if it were possible to integrate RFID devices with glass substrates in one technological process, it would significantly contribute to the development of the IoT in the scope of photovoltaic installations. The solution would become even more important if it could be used at all stages of the PV product’s life. 

**Figure 3:**
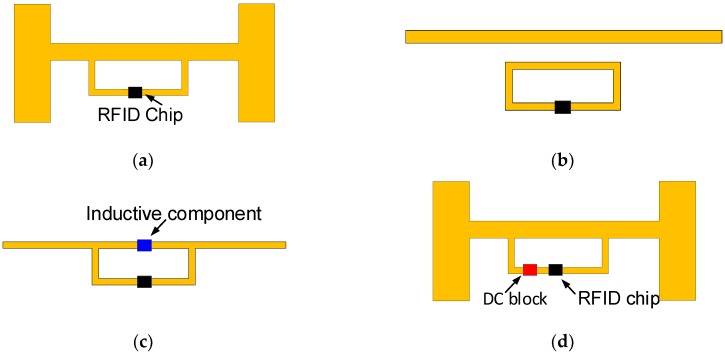
Antenna and chip impedance matching: (**a**) type T; (**b**) parasitic inductive loop; (**c**) inductive passive components; (**d**) DC block.

After choosing the antenna design, it is necessary to predict the method of its matching to the chip impedance. According to the subject literature and analysis of constructions that are available on the commercial market, the impedance matching is performed using a T-type circuit (Figure 3a) or a parasitic induction loop (Figure 3b) [1,43]. Sometimes, discrete passive components, e.g., coil attached to one of antenna arms, are used for this purpose (Figure 3c). These methods are useful in the case of passive chips and can be implemented in a wide frequency range while maintaining their uncomplicated matching structures. 

The matter gets complicated if the semipassive chips are implemented in transponders, which is the case when designing the RFID sensors for the PV applications. Such a chip (e.g., SL900A, AMS AG, Premstaetten, Austria) includes an integrated energy harvesting block that can provide energy to external electronic circuits [44]. This block is connected, inside the semiconductor structure, to the antenna RF inputs of the IC. For this reason, the semipassive chip cannot operate with the matching circuit in the form of short-circuited loop (Figure 3a). 

Therefore, the construction with open arms that are matched to the input impedance seems to be a good approach. However, such an antenna causes a number of problems, especially when is designed for use on a metal object. Its impedance-matching is only possible in a narrow frequency band (e.g., 865–868 MHz). Even relatively small inaccuracies in the geometrical dimensions of the radiator (e.g., caused by parameter deviations of antenna manufacturing process) that may be reflected in changes of the impedance value can significantly detune the RF circuit.

For aforementioned reasons, the so-called DC block (Figure 3d) is selected as the best choice for the matching impedance in the transponder/sensor intended to be integrated with the PV panels. The DC block is just a capacitor that prevents the flow of a DC signal component. The capacitor value has to be adjusted to the antenna inductance and connected to one of the antenna arms. Due to the use of this element, the energy harvester of the semipassive chip can operate despite connecting the short-circuited construction of the antenna. 

### 2.3. Antenna Design for RFID Sesnor Dedicated to PV Panels

Since the complex impedance of the antenna varies with the environmental conditions, it is very important to precisely designate material properties of the substrate to which it is attached [45,46]. The relative permittivity *ε_r_* and dielectric loss tg*δ* are the most important parameters that have to be determined for materials that are used in the PV panel constructions and that can potentially form the substrate of the transponders. 

**Figure 4:**
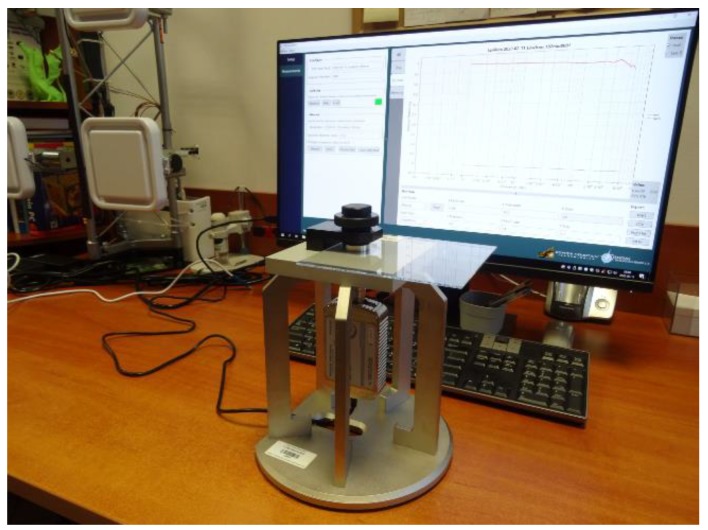
Laboratory stand of complex permittivity measurement.

In the proposed application, the low-iron glass for solar panels (thickness of 1.92 mm, measured by the universal table with the digital micrometer head Gimex GmbH 504.131) was chosen as the substrate for the designed RFID sensor. The complex permittivity of the glass was measured at the frequency of 1 GHz that is close to the operating band of UHF RFID system. This was performed in the laboratory stand composed of Compass Technology Epsilometer with integrated Copper Mountain R60 1-Port 6 GHz Vector Network Analyzer (Figure 4). The relative permittivity was equal *ε_r_* = 6.6 and the dielectric loss tg*δ* = 0.0132 at *f*_0_ = 1 GHz. 

Since the transponder is based on the semipassive SL900A chip, a few requirements determined by the authors had to be involved in the antenna design. The use of short-circuited constructions gives an opportunity to match the impedance over a wide frequency range (860–960 MHz). In addition, this solution is more resistant to the environmental conditions, such as the presence of metal frame, glass substrate and a layer of transparent conductive oxides (TCO) [43]. 

**Figure 5:**
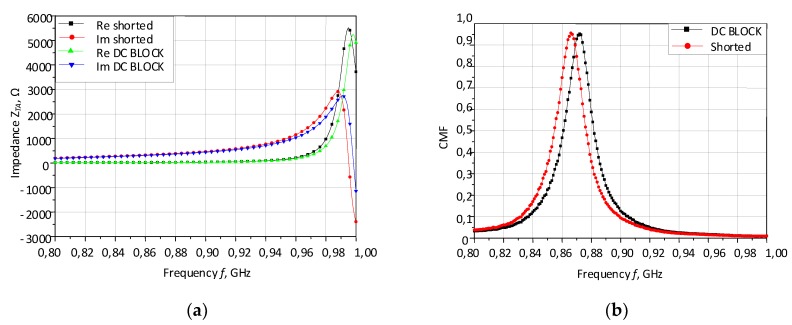
Preliminary simulation results of DC block impact. (**a**) Real and imaginary part of complex impedance. (**b**) CMF—conjugate match factor; DC BLOCK—antenna with matching circuit based on capacitor; shorted—antenna with short-circuited arms.

The DC block had to be used in order to provide the possibility of chip and antenna impedance-matching. It is recommended to choose MultiLayer Ceramic Capacitors (MLCCs) for applications that operate in the area of radio frequencies. Usually, the MLCC capacitors with a value of 33–68 pF are selected in the range 860–915 MHz but with sufficiently large *Q* factor. Taking into consideration all the described requirements and commercially available discrete components within preferred series, the MULTICOMP MC0603N560J500CT (Farnell, Leeds, UK) capacitor with a capacity of 56 pF in a 0603 housing was chosen. Preliminary simulations were carried out in order to check how the selected DC block influences the antenna impedance. The calculation results for the impedance and CMF of antennas with the DC block and the short-circuited arms are presented in Figure 5. 

A slight influence of the capacitor on the antenna impedance can be observed in the graphs (Figure 5a). The differences may be due to simplifications that are assumed in the numerical model. Instead of the gap in the antenna wire, the new port that corresponds to the DC block is defined. Moreover, the capacity value is selected from the available preferred series and slightly differs from the value calculated for the frequency of 866 MHz. 

**Figure 6:**
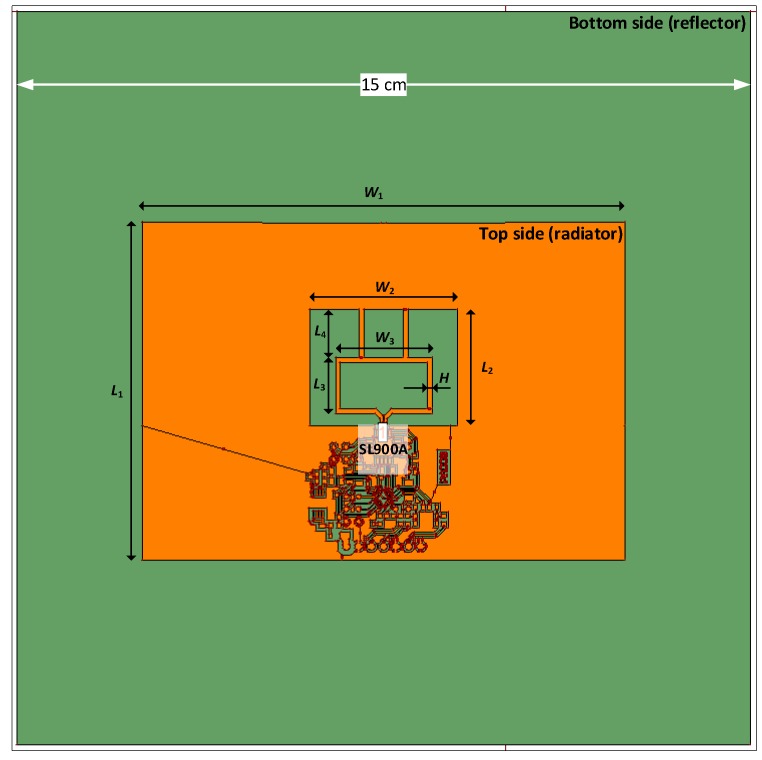
HL3DEM numerical model of antenna proposed for RFID sensor.

The antenna pattern is an example of a slot antenna in which the impedance is additionally matched using a T-shape component (Figure 6). The electronic circuits of chip and acquisition system as well as the whole antenna structure are surrounded by a relatively large ground surface in order to limit the impact of the reflector on the impedance parameters. In turn, the reflector (bottom layer) is used to reduce the influence of the internal structure of PV panel on the antenna. Generally, the antenna gain is fixed mainly by its sides *L_1_* and *W_1_* (Table 1) [43]. It can be noticed that when the width of slot *W_2_* is nearly two times smaller than the side *W_1_*, the input reactance varies slowly with the frequency, and then the antenna can be easily tuned to the chip. The impedance matching can be adjusted by changing the ratio of the slot sizes *L_2_* and *W_2_*. The resistance is sensitive mainly to the width of slot, but the reactance is dependent on both dimensions. As *W_2_* increases, the resonance moves towards lower frequencies, the resistance decreases and the reactance peak is smaller. It is also known that it is difficult to match the *Z_TA_* and *Z_TC_* impedances by using single T-match layout. Thus, by applying multiple T-match stages, further degrees of freedom can be added. The additional impedance-matching can be reached by modifying the parasitic inductive loop sizes *L_3_*, *W_3_* and *H*. This helps to improve the antenna in minimization aspect and allows designing patterns for multiband systems. The model of antenna has been elaborated in the Hyper Lynx 3D EM (HL3DEM, Mentor Graphics, Wilsonville, OR, USA). 

**Figure 7:**
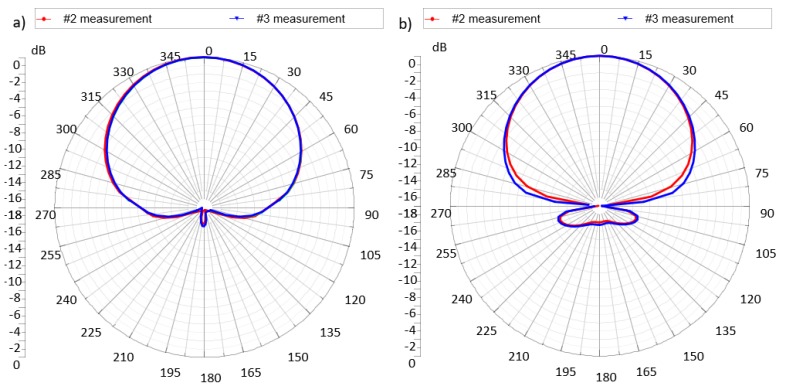
Preliminary tests of the radiation patterns.

Due to the fact that the reflector (Figure 6) with a high conductivity is placed under the radiator surface, the radiation pattern of the antenna is very strongly directional. Almost complete reduction of side and back lobes is reached, and all energy is radiated in the main lobe. The preliminary tests of the radiation patterns are presented in Figure 7. At this stage of the investigation it should be only noticed that, due to the internal construction of PV panel, an antenna design with the main lob of radiation pattern directed outside the device is advisable. By adding the reflector under the antenna, the influence of panel components is eliminated and the directionality of the antenna is ensured. Regardless of the half power beam width (HPBW), the energy should be radiated in the direction of the RWD antenna position. The fact is that the transponder can be read with a greater distance the more directional this characteristic is. However, any deviation from the designed axis of antenna system causes a sharp decrease in the read range. Therefore, both small and large values of the HPBW have their advantages and disadvantages. The analysis of radiation patterns would be interesting, but it is not very important until the application requirements are specified. Of course, this problem is of fundamental importance for determining IZ.

The impedance matching in the elaborated design was investigated for the SL900A chip from ASM corporation, enclosed in QFN16 package. This is an EPC Global Class 3 chip standardized by the ISO/IEC 18000-63 with additional custom commands (for creating extended functions of RFID sensors). The chip impedance *Z_TC_* (at power *P_Tmin_*) was obtained on the basis of the method detailed described in [10] and is specified in Table 2. It can be concluded that the impedance value is independent of the use of a supplementary battery source. This fact has essential practical meaning because it allows designers to create only one pattern of the transponder antenna for both passive and semipassive operating modes.

### 2.4. Technology of Antenna Fabrication

One of the main goals of the research is to prepare the antenna in the process that is common for manufacturing PV panels, thus allowing the tag to be easily integrated with the marked product at the fabrication line and making it capable of surviving all stages of the product life. In this scope, screen-printing is a mature process that is very often used in the technology of PV panel manufacturing. Different kinds of metallization layers are applied in this way to the constructional components of solar cells [47] (Figure 2b–d). For example, it can be implemented to create the gridlines and bus bars at the front-side glass facing the sun. The tabbing layer of silver or silver-aluminum are also printed on the rear-side of the panel constructional surface to prepare the back conductive net for connecting cells. The screen-printing process is a mature technology that can be used to obtain a variety of conductive layers with different consistency and thickness, but many parameters of the process have to be controlled in order to maintain the manufacturing repeatability, including viscosity, rheology and evaporation rate of pastes; attack angle, print speed and press setup of squeegee; mesh count; diameter; emulsion thickness of screen; and temperature and humidity in the environment. 

The measuring samples of the antenna demonstrator were manufactured in the Research & Development Centre for Photovoltaics at the ML SYSTEM Company (Rzeszów, Poland). They were prepared by applying AG1616-77 (Johnson Matthey, London, UK) conductive silver paste for thin-film coated glass (77% Ag content, ±81% firing residue) suitable for lamination applications used in the PV panel production. At the preparatory stage the mesh count, squeegee pressure, emulsion-on-mesh thickness, ink viscosity, etc. were adjusted to the experiment. Averaged values of thickness and sheet resistance obtained at the manufacturing preparatory stage were included in the MES model. 

**Figure 8:**
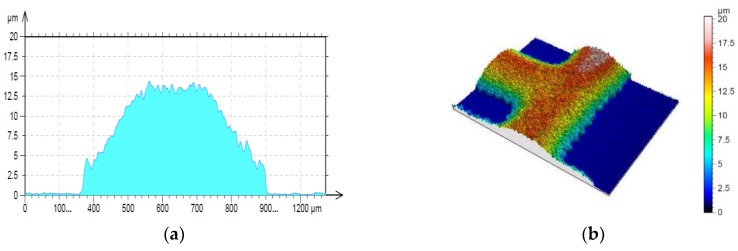
Profile of antenna elements and routes of electronic circuits: (**a**) cross-section; (**b**) surface topography.

**Figure 9:**
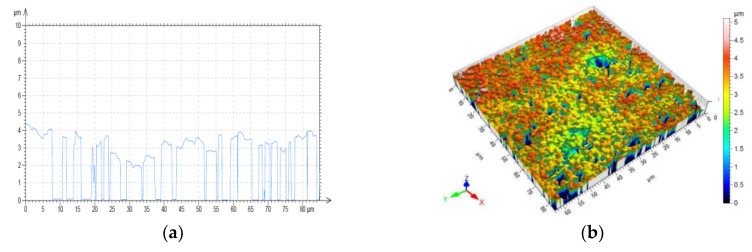
Surface roughness of conductive layer: (**a**) cross-section; (**b**) surface topography.

The samples were made out of low-iron glass with the thickness of 1.92 mm. The paste was screen-printed through a 195-mesh polyester screen, allowed to settle for 10 min and then dried at 120 °C in a heating chamber for 10 min. Subsequently the pattern was fired in a conventional furnace at 565 °C. The obtained thickness of the prepared conductive layer was around 8 µm. It was averaged on the basis of the determined profile (Figure 8) and the surface roughness (Figure 9) measured by using Sensofar optical profilometer with 20× magnification and using confocal mode. The sheet resistance of the conductive layer of the radiator (top layer—orange in Figure 6) and reflector (bottom layer—green in Figure 6) was measured by a four-probe method. The average value was equal to 17.1 × 10^6^ S/m. The parameters were determined on the basis of test results performed on the technological line in the ML System Company. 

## 3. Tests of Antenna

**Figure 10:**
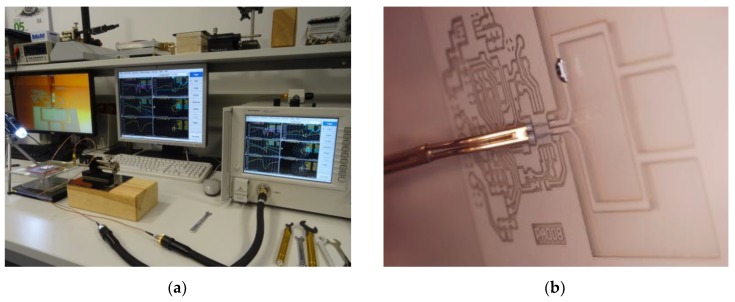
Laboratory site: (**a**) measurement stand for impedance parameter determination; (**b**) differential probe with sharp tips.

The basic antenna impedance parameters for the given type of the RFID transponders were evaluated in tests (Figure 10). Results calculated using the prepared numerical model were compared with the measurement data averaged for three samples. They were determined in the frequency band of 500–1200 MHz. 

In the measurement stand, the impact of the apparatus was restrained as much as possible. Since connectors (SMA, N, UFL, etc.) are not used in the transponders, they also should not be used at the laboratory side because they significantly interfere the balance of the impedance in the antenna resonance circuit. Thus, the differential passive probe with signal-to-signal delicate contact tips was used to connect the antenna to the vector network analyzer with two 50 Ω coaxial ports. The measure procedure—described in detail in [9]—constitutes in the S-parameters determination:

(4) $$Z_{d}=\frac{2Z_{0}\left(S_{12}S_{21}-S_{11}S_{22}-S_{12}-S_{21}+1\right)}{\left(1-S_{11}\right)\left(1-S_{22}\right)-S_{12}S_{21}}$$

where *Z*_0_ denotes the reference impedance of 50 Ω. The laboratory stand included VNA Keysight PNA-X N5242A and a commercially available probe (Micromanipulator 44-8000-D-NA) which was moved using a manipulator (Micromanipulator model 110) with three-axis direct lead-screw/lead-nut drivers of resolution 2.2 µm (10 mm max range in each axis). The set of Keysight 85131F flexible cables (3.5 mm) was used to connect the probe and VNA.

**Figure 11:**
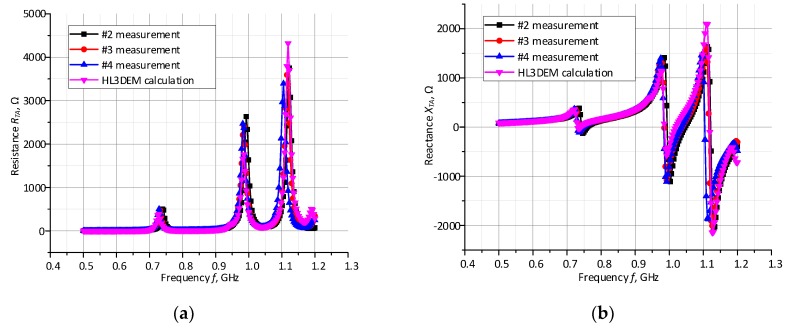
Comparison of measurements and numerical calculations of antenna complex impedance *Z_TA_*: (**a**) real part; (**b**) imaginary part.

The antenna was matched to the chip impedance (14.6−j 316) Ω, with a typical resistance value Re(*Z_TC_*) in the range from a few to tens of Ohms and a reactance value Im(*Z_TC_*) of about a few hundred of Ohms (at the chip sensitivity *P_Tmin_,* in the frequency band of *f*_0_ = 860–960 MHz). In Figure 11, it is possible to find a high correlation between calculations and measurements performed for all test samples in the frequency band 500–1200 MHz. Since the Pearson correlation coefficient between the average of samples 2–4 and the HL3DEM calculations is equal 0.918 for the real part and 0.911 for the imaginary part, the obtained results of the impedance *Z_TA_* show the satisfactory convergence which is suitable for analyzing the impedance matching between the antenna and selected chip in the whole RFID UHF band (860–960 MHz).

**Figure 12:**
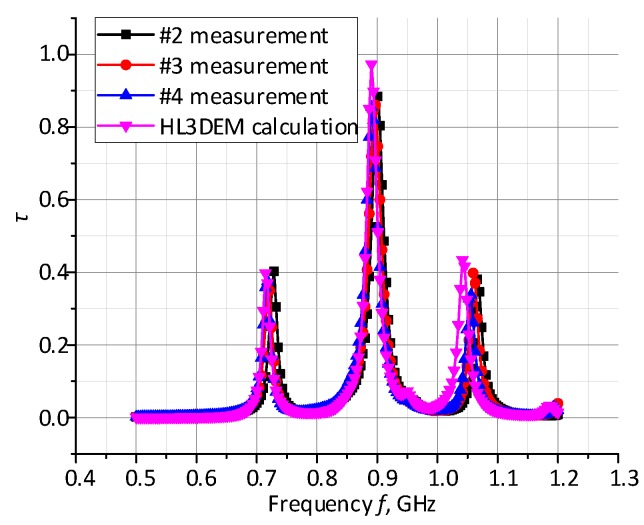
Comparison of measurements and numerical calculations of power transfer coefficient.

The comparison of measurements and numerical calculations of power transfer coefficient presented in Figure 12 also confirms correct operation of the designed antenna. 

## 4. Conclusions

In this work, the uncommon construction of the RFID antenna dedicated to being fabricated on a cover glass of PV panels is elaborated. Detailed information on the design process and restrictions that should be taken into account is disclosed in the paper. The antenna is designed to be manufactured in the screen-printing process that is typically used in producing photovoltaic cells. It can cooperate with the semipassive chip that can be implemented as a key component of the RFID sensor. The antenna is designed in the shape that allows for easy access to its contacts when an electronic circuit of measured parameter acquisition system needs to be connected.

The numerical model of the antenna was elaborated in the HL3DEM software tool, and test samples were fabricated on the technology line of ML System Company in the screen-printing process of pastes on a glass substrate. The high correlation between calculations and measurements performed for all test samples in the frequency band of 500–1200 MHz confirms the proper course of the design. The obtained results of the impedance *Z_TA_* in the 860–960 MHz frequency band show a satisfactory convergence which is suitable for analyzing the impedance-matching between the antenna and the selected SL900A chip.

## Figures and Tables

**Table 1:** Dimensions of designed antenna in accordance with designations in Figure 6.

Specimen Geometry, Dimensions, mm							
*L*_1_	*L*_2_	*L*_3_	*L*_4_	*W*_1_	*W*_2_	*W*_3_	*H*
72.10	26.80	13.90	11.90	98.80	30.00	19.70	1.00

**Table 2:** Measuring results of impedance for passive/semipassive SL900A chip.

Frequency, MHz	*Z_TC_*, Ω	*Z_TC_*, Ω
	Passive Mode	Semipassive Mode
	*U_bat_* = 0 V	*U_bat_* = 1.5 V
*f*_0_ = 866	14.9−j 342	14.7−j 338
*f*_0_ = 900	14.9−j 342	14.4−j 316
*f*_0_ = 915	15.3−j 313	14.9−j 309
mean value => 860–960	14.5−j 316	14.0−j 314
